# A novel moisturizer with high sun protection factor improves cutaneous barrier function and the visible appearance of rosacea‐prone skin

**DOI:** 10.1111/jocd.12889

**Published:** 2019-02-25

**Authors:** Hilary Baldwin, Francine Santoro, Nadege Lachmann, Sandrine Teissedre

**Affiliations:** ^1^ The Acne Treatment and Research Center Morristown New Jersey; ^2^ Galderma Research and Development Egerkingen Switzerland; ^3^ Galderma Research and Development Sophia Antipolis France

**Keywords:** barrier function, moisturizer, rosacea, sun protection factor

## Abstract

**Background:**

Consensus guidelines advocate general skincare for rosacea patients.

**Objectives:**

Two independent studies were performed to assess whether a tinted daily SPF‐30 facial moisturizer (DFM30) improves barrier function of dry skin and the efficacy and tolerability of DFM30 on rosacea‐prone skin.

**Methods:**

In study 1, electrical capacitance (EC) and transepidermal water loss (TEWL) were measured at baseline, 2, 4, 8, and 24 hours after a single application of DFM30 and on a control site in 21 healthy females with dry skin. Study 2 evaluated 33 females with mild to moderate rosacea and nontransient erythema. Efficacy and tolerability after once‐daily DFM30 were assessed using a chromameter, image analysis of photographs, and trained rater and patient evaluations up to day 22.

**Results:**

In study 1, EC showed statistically significant increases at 2, 4, and 8 hours, and TEWL showed statistically significant decreases 2, 4, 8, and 24 hours after DFM30 application to healthy females compared to baseline. In study 2, covering skin redness improved significantly after DFM30 application on day 1; 33.3% showed improved covering skin redness compared to baseline. Patients reported significantly less redness on day 8 than day 3. Feelings of dryness and tightness/tension were lower 30 minutes after first application. Feeling of dryness was lower than baseline after 3 days, 1 and 3 weeks. Image analysis suggested redness was significantly lower on day 22 compared to baseline. Chromameter readings showed significantly lower erythema on the cheek compared to baseline. All patients stated that DFM30 relieves and neutralizes visible redness who also indicated that they would purchase DFM30, and the product was well tolerated.

**Conclusions:**

These studies show that DFM30 is suitable as part of the skincare regimens advocated by ROSacea COnsensus (ROSCO) for rosacea patients. DFM30 is an effective moisturizer that improves cutaneous barrier function and the appearance of rosacea‐prone skin.

## 1 INTRODUCTION

Approximately 5%‐10% of the population suffers from rosacea, a chronic inflammatory skin disorder.^1^, ^2^ The chronic inflammation that underlies rosacea can result in a variety of signs and symptoms that include flushing, telangiectasia, inflammatory lesions, and ocular manifestations.^3^ Two features, however, seem to be diagnostic of rosacea: firstly, persistent erythema that affects the central face and that shows periodic intensification and, secondly, phyma, especially affecting the nose.^3^ As rosacea affects facial appearance, the condition can have a marked psychological impact and undermine health‐related quality of life.^4^, ^5^, ^6^, ^7^

Against this background, the global ROSacea COnsensus (ROSCO) panel recommended tailoring treatment according to the phenotype (eg transient and persistent erythema, inflammatory papules and pustules, telangiectasia and phyma). In addition, all rosacea patients should be educated on good general skincare, which is the main strategy to manage secondary features, such as dry appearance, dry sensation, and stinging sensation.^8^

The ROSCO guidelines stress that skincare regimens should include using sunscreen (sun protection factor [SPF] ≥30).^8^ Between 61% and 81% of patients with rosacea cite sun exposure as a contributory factor.^4^, ^9^ Skincare suggested by ROSCO include gentle cleansers, avoidance of triggers, and frequent application of quality moisturizers.^8^ Rosacea patients often experience dry facial skin that can exacerbate symptoms.^10^ Moisturizers can repair and maintain stratum corneal barrier function, enhance skin hydration, and reduce the likelihood of skin irritation. As a result, moisturizers relieve dry skin, improve softness and suppleness, and can be adjuvants to other rosacea therapies.^10^

This paper reports the results of two independent studies into the use of a novel tinted daily SPF‐30 facial moisturizer (DFM30), designed according to the new ROSCO guidelines, as part of the skincare regimen for rosacea patients. One study aimed to determine whether DFM30 improves barrier function in otherwise healthy women with dry skin. The other study assessed the efficacy and tolerability of a tinted daily facial moisturizer with an SPF of 30 (DFM30) in patients presenting with rosacea‐prone skin.

## 2 MATERIALS AND METHODS

Two independent studies were conducted in accordance with the Declaration of Helsinki, Good Clinical Practice, and local regulatory requirements.

The first study was performed in conformity with the standard Operating Procedures of Institut d’ Expertise Clinique. The second study adhered to the protocol and compliance with the quality system of ProDerm. Audits were performed at regular intervals to verify adherence to both study protocols.

In both studies, room humidity and temperatures were maintained within published guidelines^11^ and patients or healthy women rested in the room for at least 30 minutes before measurements. Safety was monitored by reporting of adverse events.

### 2.1 Study A (cutaneous barrier function)

The first study (performed at IEC France, Lyon) enrolled 21 healthy Caucasian females (age 18‐70 years) with dry skin on their inner forearm and an initial moisturization level that corresponded to electrical capacitance values of ≤50 arbitrary units.

Patients with rosacea often present with a defective skin barrier and therefore an increased transepidermal water loss (TEWL).^12^, ^13^ Healthy subjects with dry skin were used in order to “mimic” the damaged barrier and increased TEWL that is found in patients with rosacea.^12^, ^13^

Dryness was defined with an objective dermatological assessment of dryness as well as subjective reports from the participants. TEWL was measured using a Tewameter TM 300 (Courage & Khazaka) and electrical capacitance using a Corneometer CM 825 (Courage & Khazaka). Values for electrical capacitance are given in arbitrary units (scale from 0 to about 130), which reflects the degree of moisturization in the upper layers of the epidermis.

The test and control areas were 20 cm^2^ areas of dry skin on the inner forearm. A technician applied DFM30 (2 mg cm^−2^) to the test area on the right or left forearm according to randomization and massaged until the product penetrated completely. Electrical capacitance and TEWL were measured at baseline and 2, 4, 8, and 24 hours after a single application of DFM30.

Descriptive statistics summarized the results, and differences were assessed using the Shapiro‐Wilk test (significance defined as *P* < 0.01). Treated and control areas were compared using the Student *t* test or Wilcoxon test (both two‐tail, significance defined as *P* < 0.05) for normal and nonparametric distributions, respectively.

### 2.2 Study B (efficacy and tolerability)

The second study (performed at the proDERM Institute for Applied Dermatological Research GmbH, Schenefeld/Hamburg) planned to enrol 33 female or male patients with type I rosacea presenting with mild to moderate nontransient erythema. The study aimed to enrol at least 50% of patients with self‐reported sensitive skin. Eligible patients were between 25 and 75 years of age, with a maximum of 20% of the cohort being over 60 years. The study aimed to enrol approximately 25% of patients with Fitzpatrick type I skin, approximately 50% of patients with Fitzpatrick type II, and approximately 25% of patients with Fitzpatrick type III.

Chromameter (CR 300 or CR 400; Minolta, Device D‐Langenhagen, Germany) measurements were performed on the right cheek and on unaffected forehead, and a full‐face image was taken using VISIA‐CR BOOTH (Canfield Clinical Systems, Fairfield, NJ), which offers standardized, computer‐controlled facial photography. Image analysis of skin color (a*‐value, which corresponds to an increase in the degree of skin redness) was performed on the images using cross‐polarized light.

A trained technician ensured that patients applied DFM30 correctly and assessed tolerability (scaling, fissures, papules, pustules, edema, vesicles, weeping). Patients rated their agreement with statements about efficacy and traits immediately after application. Statements are shown in the results section. Redness, subjective efficacy (tension/tightness, feeling of dryness), subjective tolerability (itching, burning, tickling), and subjective eye status (itching, burning, sand grain feeling) were assessed, and the full‐face images were repeated 30 ± 5 minutes after DFM30 application.

Patients applied DFM30 once daily in the morning at home, according to normal use‐conditions. Patients returned to the study site on days 3, 8, and 22, when chromameter measurements were performed. A deviation of ±2 days on day 22 was permitted. Dryness was assessed by the technician on days 8 and 22. Skin redness was assessed by a trained rater and by the patients on days 3, 8, and 22. Patients assessed subjective efficacy on these days. Subjective tolerability, technician‐evaluated tolerability, and eye status were assessed on day 22. In addition, on day 22, a full‐face image was taken and patients rated their agreement with statements about product efficacy and traits.

Trained raters and patients assessed skin redness or covering of skin redness using a five‐point scale ranging from “None” to “Strong.” Raters assessed skin dryness and objective skin status (scaling, fissures, papules, pustules, edema, vesicles, weeping) using this five‐point scale. Patients used the same five‐point scale to evaluate subjective efficacy, tolerability, and eye status, and rated their agreement with statements about product efficacy and traits using a five‐point scale ranging from “Fully agree” to “Fully disagree.”

Statistical significance was defined as *P* < 0.05. Rater‐assessed outcomes were compared using Wilcoxon signed‐ranks test. McNemar's test was used for worsened and improved assessments. The Binomial test was used for frequencies of answers between the “agree” and “disagree” groups. Instrumental measurement parameters were compared using a paired *t* test.

## 3 RESULTS

### 3.1 Study A (cutaneous barrier function)

Thirty healthy females with dry skin on their inner arm were screened, of which 21 were eligible (mean age 46.0; range 20‐68 years). All those enrolled completed the study.

**Figure 1:**
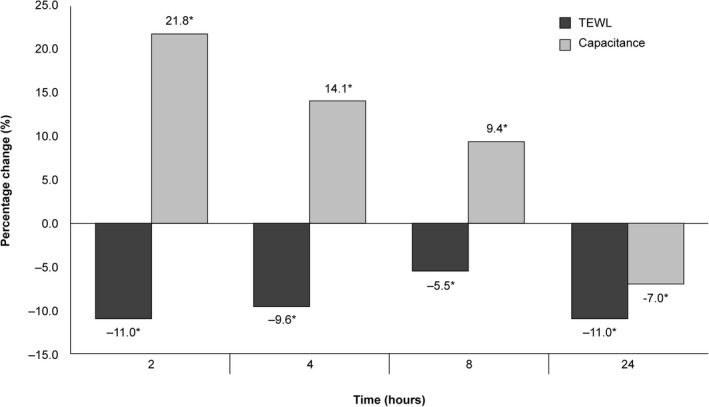
Variation in TEWL and electrical capacitance following a single application of DFM30. * indicates a statistically significant difference. Percentages indicate the change in electrical capacitance and therefore moisturization level. DFM30, daily SPF‐30 facial moisturizer; TEWL, transepidermal water loss

Figure 1 and Table 1 summarize the results. There was no statistically significant difference in electrical capacitance or TEWL between control and treated areas at baseline. Electrical capacitance showed statistically significant increases 2, 4, 8, and 24 hours after DFM30 application compared to baseline. TEWL showed statistically significant decreases 2, 4, 8, and 24 hours after DFM30 application compared to baseline.

**Table 1:** TEWL and electrical capacitance following a single application of DFM30

	Mean ± SD		*P* value
	Control area	DFM30 area	
TEWL (g m^−2^ h^−1^)			
Initial measurement (T0)	7.3 ± 1.4	7.3 ± 1.3	0.928
2 h	7.1 ± 1.5	6.3 ± 1.4	—
4 h	7.3 ± 1.5	6.6 ± 1.4	—
8 h	7.3 ± 1.4	6.9 ± 1.4	—
24 h	7.3 ± 1.4	6.5 ± 1.3	—
Difference between 2 h and T0	−0.2 ± 0.7	−1.0 ± 1.0	0.03
Difference between 4 h and T0	0.0 ± 0.7	−0.7 ± 1.0	0.001
Difference between 8 h and T0	0.0 ± 0.6	−0.4 ± 0.9	0.042
Difference between 24 h and T0	0.0 ± 0.7	−0.8 ± 0.8	0.003
Electrical capacitance (arbitrary units)			
Initial measurement (T0)	30.0 ± 7.2	29.8 ± 6.5	0.706
2 h	29.9 ± 6.7	36.2 ± 6.5	—
4 h	29.8 ± 6.5	33.8 ± 5.9	—
8 h	30.1 ± 7.2	32.7 ± 5.3	—
24 h	31.4 ± 7.1	29.1 ± 5.4	—
Difference between 2 h and T0	−0.1 ± 2.6	6.4 ± 3.3	<0.001
Difference between 4 h and T0	−0.3 ± 2.3	4.0 ± 4.0	<0.001
Difference between 8 h and T0	0.0 ± 2.5	2.9 ± 3.6	0.002
Difference between 24 h and T0	1.3 ± 2.6	−0.7 ± 3.6	0.01

DFM30, daily SPF‐30 facial moisturizer; TEWL, transepidermal water loss.

### 3.2 Study B (efficacy and tolerability)

Thirty‐three females (mean age 53.7 ± 10.4 years) were enrolled of which three did not complete the study for reasons that were not associated with DFM30. Thirty patients self‐reported having sensitive skin.

No statistically significant differences were found for skin redness (Table 2) assessed by a trained rater at any time (ie baseline vs day 3, 8 or 22; day 3 vs day 8 or 22; day 8 vs day 22). However, covering skin redness assessed by a trained rater improved significantly 30 minutes after application on day 1 (*P* = 0.004) compared to baseline. A third (33.3%) of patients showed an improvement in covering skin redness (*P* = 0.002) compared to baseline.

**Table 2:** Efficacy of DFM30

Time	None (%)	Very slight (%)	Slight (%)	Moderate (%)	Strong (%)
Skin redness assessed by trained rater					
Baseline	0.0	23.3	46.7	30.0	0.0
Day 3	0.0	16.7	60.0	23.3	0.0
Day 8	0.0	16.7	56.7	26.7	0.0
Day 22	0.0	20.0	43.3	33.3	3.3
Covering skin redness assessed by trained rater					
Baseline	0.0	23.3	46.7	30.0	0.0
Day 1 30 min PA	0.0	56.7	33.3	10.0	0.0
Skin redness assessed by patient					
Baseline	0.0	3.3	50.0	43.3	3.3
Day 3	0.0	10.0	33.3	46.7	10.0
Day 8	0.0	13.3	50.0	36.7	0.0
Day 22	0.0	23.3	33.3	36.7	6.7
Covering skin redness assessed by patient					
Baseline	0.0	3.3	50.0	43.3	3.3
Day 1 30 min PA	0.0	36.7	30.0	33.3	0.0

DFM30, daily SPF‐30 facial moisturizer; PA, postapplication.

No statistically significant differences were found for skin redness (Table 2) assessed by the patient at most times (baseline vs day 3, 8 or 22; day 3 vs day 22; day 8 vs day 22). However, rosacea patients reported significantly less redness on day 8 compared to day 3 (*P* = 0.016). Patients stated that covering skin redness improved significantly 30 minutes after application on day 1 (*P* = 0.006) compared to baseline. A third (33.3%) of rosacea patients reported an improvement in covering skin redness (*P* = 0.002) compared to baseline. The feeling of dryness and tightness/tension was lower 30 minutes after the first application of DFM30. After 3 days, as well as after 1 and 3 weeks use, the feeling of dryness was lower than at baseline. The feeling of tightness/tension remained comparable to baseline throughout the study, although decreased at day 1 after 30 minutes.

Image analysis suggested that skin redness (Table 3) was significantly lower on day 22 compared to baseline on the unaffected forehead. No other significant differences were found.

**Table 3:** Skin redness assessed by image analysis and chromameter

Test area	Time	Mean value	*P* value
Skin redness assessed by image analysis (a*‐value)			
Cheek	Baseline	21.804	0.889
	Day 22	21.858	
Forehead	Baseline	16.713	0.030
	Day 22	16.154	
Difference between check and forehead	Baseline	5.091	0.064
	Day 22	5.704	
Covering skin redness assessed by image analysis (a*‐value)			
Cheek	Baseline	21.804	0.431
	Day 1 30 min PA	21.967	

Erythema measured by chromameter (a*‐value)		Versus baseline	Versus day 3	Versus day 8
Cheek				
Baseline	20.957	—	—	—
Day 3	19.168	<0.001	—	—
Day 8	19.857	0.002	0.003	—
Day 22	19.766	<0.001	0.021	0.726
Forehead				
Baseline	16.333	—	—	—
Day 3	16.279	0.851	—	—
Day 8	16.087	0.327	0.347	—
Day 22	15.899	0.198	0.066	0.416
Difference between check and forehead				
Baseline	4.624	—	—	—
Day 3	2.889	<0.001	—	—
Day 8	3.771	0.016	0.007	—
Day 22	3.867	0.078	0.007	0.786

PA, postapplication.

Chromameter readings showed significantly lower erythema at each measurement time on the cheek compared to baseline. Significantly higher a*‐values were recorded on days 8 and 22 compared to day 3 on the cheek. No significant differences were apparent on the forehead. The difference between the cheek and forehead was significantly lower compared to baseline on days 3 and 8. Significantly higher a*‐values for the difference between the cheek and forehead were recorded on days 8 and 22 compared to day 3.

After the first application of the DFM30 and on day 22, significantly more patients agreed with all statements regarding its product traits and efficacy than disagreed (Table 4). After 3 weeks, the statements that the DFM30 neutralizes the look of redness and relieves visible redness obtained highest agreement.

**Table 4:** Assessment of traits and efficacy by patients

Statement	Disagree (%)	Undecided (%)	Agree (%)	Relative frequency (%)		
				Disagree	Agree	*P* value
Day 1 directly after application						
The product has a soothing effect	6.7	16.7	76.7	8.0	92.0	<0.001
The product leaves my skin soft and smooth	0.0	13.3	86.7	0.0	100.0	<0.001
The product visibly improves my skin tone	3.3	10.0	86.7	3.7	96.3	<0.001
The product reduces the feeling of discomfort	6.7	30.0	63.3	9.5	90.5	<0.001
The product helps conceal skin redness immediately	6.7	6.7	86.7	7.1	92.9	<0.001
The product can be used as an alternative to makeup due to the tinted coverage	10.0	0.0	90.0	10.0	90.0	<0.001
The product is easy to apply	3.3	0.0	96.7	3.3	96.7	<0.001
The product has a fast absorption but coverage remains	3.3	0.0	96.7	3.3	96.7	<0.001
After product application, I immediately feel more confident due to my redness being less evident	10.0	30.0	60.0	14.3	85.7	0.001
Day 22						
The product has a soothing effect	13.3	23.3	63.3	17.4	82.6	0.003
The product leaves my skin soft and smooth	16.7	23.3	60.0	21.7	78.3	0.011
The product visibly improves my skin tone	10.0	20.0	70.0	12.5	87.5	<0.001
The product neutralises the look of redness	3.3	0.0	96.7	3.3	96.7	<0.001
The product relieves visible redness	3.3	0.0	96.7	3.3	96.7	<0.001
The product reduces the feeling of discomfort	23.3	13.3	63.3	26.9	73.1	0.029
The product is suitable for sensitive skin	6.7	20.0	73.3	8.3	91.7	<0.001
The product can be used as an alternative to makeup due to the tinted coverage	13.3	3.3	83.3	13.8	86.2	<0.001
The product was easy to incorporate into my daily skin care regimen	6.7	3.3	90.0	6.9	93.1	<0.001
The product improves skin texture	16.7	30.0	53.3	23.8	76.2	0.027
The product is easy to apply	6.7	3.3	90.0	6.9	93.1	<0.001
When I apply the product, I feel that my skin is nourished	20.0	3.3	76.7	20.7	79.3	0.002
When I apply the product, I feel more confident in my skin tone	10.0	13.3	76.7	11.5	88.5	<0.001
Overall, I like the product very much	23.3	6.7	70.0	25.0	75.0	0.013
Would you like to buy this product?	0.0	0.0	100.0	0.0	100.0	<0.001

Only single cases of itching, burning, and tickling were diagnosed by the trained rater throughout the study. This included 1 case of itching on day 1, and 1 case of burning and 1 case of tickling on day 22. Patients reported only isolated cases of “sand grain feeling” and no itching and burning. One patient developed a small exfoliative, dry area on her neck. Raters assessed the severity as mild and that there was a reasonable possibility that the event was related to DFM30. No action was taken and the patient recovered without treatment or sequelae.

## 4 DISCUSSION

The ROSCO guidelines note that education and instruction about general skin care is “essential” for all patients with rosacea to ensure the best possible treatment outcomes. Elements in skincare include sunscreen, frequent use of moisturizers and gentle over‐the‐counter cleansers, and avoiding known triggers. Indeed, general skincare is the main management strategy for the secondary features of rosacea.^8^ The two studies presented in this paper show that the DFM30 is suitable as part of the skincare regimens advocated by ROSCO for rosacea patients, enhances cutaneous barrier function, and improves the visible appearance of rosacea‐prone skin.

The first study enrolled healthy females presenting with dry skin on forearms. A single application of DFM30 produced statistically significant moisturization of the upper layers of the epidermis that persisted for 8 hours after the application. DFM30 also led to a statistically significant decrease in TEWL about 24 hours later, which is consistent with enhanced barrier function. These findings in otherwise healthy women were confirmed by clinical observations of patients with rosacea. Raters noted a reduction in dryness. Patients reported that the feeling of dryness and tightness/tension was noticeably lower 30 minutes after the first application. After 3, 8, and 22 days, the feeling of dryness was lower than baseline. Improved dryness and scaling is consistent with increased hydration of the stratum corneum.^10^ Subjects with dry skin were used in this study as patients with rosacea often present with a defective skin barrier and therefore an increased TEWL. Accordingly, subjects with dry skin on the forearms were used to replicate the damaged barrier and increased TEWL that is found in such patients.^12^, ^13^

These enhancements in skin function seem to translate into improved outcomes in patients with type I rosacea and mild to moderate nontransient erythema. DFM30 is tinted, and 30 minutes after the first application, assessments by the patient and the rater showed a camouflage effect, which was confirmed by chromameter measurements on the cheek. Image analysis, however, did not confirm a reduction in visible redness. The reasons for the discordance between the assessment methods are unclear but may be related to interference by the pigment included in the product with the measurement system.

Subjective assessment showed no reduction of skin redness after 1 and 3 weeks of application. This suggests that camouflage associated with the tinted formulation was responsible for the anti‐redness effect. However, rosacea patients reported significantly less redness on day 8 compared to day 3. Image analysis showed a significantly reduced skin redness on day 22 compared to baseline on the nonaffected forehead. As this was an exploratory study, the statistical analysis did not account for multiple observations and these may be type 1 errors.

The chromameter measurements on the cheek indicated improved erythema at all times compared to baseline. After correcting using data obtained from the control area (forehead), the results were confirmed. However, the effect on day 22 just missed significance (*P* = 0.078), suggesting a larger study may be warranted. Therefore, the study could be repeated in a larger number of patients using statistical analysis of multiple observations.

Patients showed a strong agreement with statements about favorable traits and attributes, suggesting that DFM30 would be an effective adjuvant to treatment for rosacea. Indeed, all the patients stated that they would like to buy DFM30. A well‐accepted product is likely to help ensure good adherence, especially during long‐term use, although further studies with longer follow‐up are needed to confirm this hypothesis.

Many patients with rosacea report heightened skin sensitivity with skincare and personal hygiene products^14^ and dry facial skin can exacerbate symptoms.^10^ Despite enrolling patients with skin sensitivity, the overall tolerability of DFM30 was very good, which should help ensure good adherence.

While these results show that DFM30 is effective and well tolerated, the studies had certain limitations and raised further suggestions for additional investigations. As mentioned above, the study could be repeated in a larger number of patients using statistical analysis of multiple observations. Each of the studies was performed at one center. They could be replicated in a larger, more diverse sample including men, more diverse skin types and the different phenotypes of rosacea. The benefit against UV/sun exposure could also be assessed in a “naturalistic” study, conducted in a natural setting, and the TEWL and skin conductance study replicated on patients with rosacea.

## 5 CONCLUSIONS

The two studies in this paper show that DFM30 is suitable as part of the skincare regimens advocated by ROSCO for patients with rosacea. In healthy females presenting with dry skin on their forearms, a single application of DFM30 produced a statistically and clear moisturization effect that persisted for 8 hours. DFM30 also decreased in TEWL about 24 hours later, suggesting strengthening of barrier function. This finding was confirmed by clinical observation that DFM30 improved dryness in the rosacea patients.

These improvements in skin function seem to translate into improved outcomes in patients with type I rosacea and mild to moderate nontransient erythema. DFM30 is tinted, and 30 minutes after the first application, assessments by the rosacea patients and the rater showed a camouflage effect. Patients agree that the product's traits and attributes could be appropriate for a skincare product for rosacea. All rosacea patients stated that they would like to purchase the product. Taken together, these results show that DFM30 is an effective and well‐tolerated moisturizer that improves cutaneous barrier function and the visible appearance of rosacea‐prone skin.

## CONFLICTS OF INTEREST

NL and FS are employees of Galderma R&D Egerkingen, Switzerland. HB serves as an advisor, investigator or on the speaker's bureau for Galderma, Ortho Dermatologic, Bayer, L'Oreal and La Roche‐Posay. ST is an employee of Galderma Research & Development, Sophia Antipolis, France.
